# Oral health status, self-perceptions, and risk awareness among young adult users of electronic cigarettes in Pakistan

**DOI:** 10.18332/tid/194963

**Published:** 2024-11-05

**Authors:** Osama Khattak, Farooq A. Chaudhary, Asma Sakoor, Muhammad U. Khattak, Aliya Ehsan, Farida Habib Khan, Ayesha A. Khalid, Yasir D. Siddiqui, Azhar Iqbal, Rakhi Issrani

**Affiliations:** 1 Department of Restorative Dentistry, College of Dentistry, Jouf University, Sakaka, Saudi Arabia; 2 School of Dentistry, Shaheed Zulfiqar Ali Bhutto Medical University, Islamabad, Pakistan; 3 Institute of Dentistry, CMH Lahore Medical College, National University of Medical Sciences, Rawalpindi, Pakistan; 4 Department of Periodontology, Islamic International Dental College, Riphah International University, Islamabad, Pakistan; 5 Department of Family and Community Medicine, College of Medicine, University of Hail, Saudi Arabia; 6 Cardiology Department, The Hillingdon Hospital, Uxbridge, United Kingdom; 7 Department of Preventive Dentistry, College of Dentistry, Jouf University, Sakaka, Saudi Arabia

**Keywords:** oral health, young adults, electronic cigarette, clinical oral status, self-perceived dental status

## Abstract

**INTRODUCTION:**

The use of electronic cigarettes (ECs) has surged globally, particularly among young individuals. This study aimed to assess the perceptions of vaping-related oral health risks, clinical oral health status, and self-perceived dental and periodontal conditions among young adult users of ECs in Pakistan.

**METHODS:**

A cross-sectional study was conducted from June 2023 to March 2024, recruiting 142 young users of ECs. Intraoral examinations assessed Decayed, Missing, and Filled Teeth (DMFT) index, Oral Hygiene Index Simplified (OHI-S), Gingival Bleeding Index (GBI), Plaque Index (PI), and dental stain. Data on sociodemographic characteristics, oral health behaviors, vaping habits, and perceptions of impact of vaping on oral health were gathered through a self-administered questionnaire. Associations between EC use and various oral health variables were analyzed using the chi-squared and Fisher’s exact tests.

**RESULTS:**

Mean DMFT was 5.66 (SD=2.20). Poor oral hygiene (29.6%) and severe dental staining were prevalent. Most participants (76.1%) brushed once daily, while only 34.5% attended regular dental check-ups. Gingival bleeding and plaque accumulation were observed in 47.2% and 35.3% of participants. Around 66% reported daily EC use, with 80.3% initiating vaping before the age of 18 years. Most common reason for vaping was perception that ECs are safer than traditional smoking (31.7%). Participants' perceptions of vaping-related oral health risks were relatively low, with 45% associating vaping with tooth decay, 48% with gum disease, and 58.5% with tooth staining. Tooth brushing frequency, vaping frequency (per day), and time since vaping started, were significantly associated with oral clinical indicators (p<0.05). The education level was the only variable significantly associated with vaping-related risk perception (p<0.05).

**CONCLUSIONS:**

The study reveals that oral health awareness among young vapers is low, with many starting EC use at a young age and exhibiting poor oral health behaviors. Misconceptions about the safety of ECs compared to conventional cigarettes may contribute to increased vaping.

## INTRODUCTION

Electronic cigarettes (ECs) or vapes are handheld devices powered by batteries, containing an e-liquid, which typically includes nicotine, various chemicals such as formaldehyde and acetaldehyde, and occasionally artificial flavors like menthol, coffee, candy, butter, and various fruits^1,2^. This liquid is heated by a heating element, producing a chemical-filled aerosol that can be inhaled. The prevalence of electronic cigarette (EC) or vaping usage among young individuals has surged worldwide, particularly in areas where these products are readily available. Findings from a survey conducted in the United States revealed that nearly one in ten individuals who had never smoked had tried vaping e-cigarettes at least once^3^. Moreover, the survey indicated that e-cigarette usage is more prevalent among younger adults (aged 18–24 years) than among older adults (aged ≥65 years), with males exhibiting a higher prevalence compared to females^3^. Initially ECs were marketed as a tool to help people quit smoking, but now they are also used for social enjoyment and their usage rapidly surged to epidemic proportions in certain parts of the world^4^. Despite the misconception that e-cigarette vaping is less harmful than traditional tobacco smoking, recent studies have demonstrated that e-cigarette aerosol induces oxidative stress, inflammation, and disruption of lung cellular function^5^. This includes impairments in myofibroblast differentiation, mitochondrial stress, and the promotion of DNA fragmentation and contributing to cardiovascular, respiratory, pulmonary and oral diseases^6^.

Pakistan is emerging as a major vaping and e-cigarette business hub, as currently it has no regulations or restrictions in place regarding the sale, use, promotion, sponsorship, and advertising of ECs or vaping^7-9^. It is estimated that the revenue generated from e-cigarettes in Pakistan will reach $77.2 million by 2024, experiencing an annual growth rate of 1.39%^7^. The trend of vaping has been steadily increasing in popularity in Pakistan over the past few years. In 2017, a cross-sectional study carried out in Karachi, Pakistan, uncovered that numerous participants were utilizing vaping devices without a comprehensive grasp of their contents and the detrimental impacts they could have^10^.

Investigation of the impact of vaping on oral health is starting to reveal evidence suggesting that the nicotine and other chemical compounds present in electronic cigarette liquids and vapor might be linked to oral health complications^11^. Periodontal damage and irritation of the mouth and throat are the most frequently reported consequences^12,13^. While numerous studies have explored people’s understanding and attitudes regarding the impact of vaping on overall health, only a few have explored the perceived risk of vaping specifically on individual oral health. Currently, it remains uncertain how young individuals in Pakistan perceive the potential adverse effects on both their overall and oral health. Oral health stands out as one of the most overlooked aspects of health for young people, despite its financially and socially detrimental impacts^14^. Presently, to our knowledge, there is limited research available describing the perception of young people regarding the risks of vaping on their oral health in Pakistan. The aims of this study were: 1) to understand the perceptions of vaping and its associated oral health risks among young individuals, 2) to evaluate the oral health status and self-perceived dental and periodontal status of electronic cigarette smokers, and 3) to investigate the association between self-assessed oral and periodontal health and the usage of electronic cigarettes, among young adults in Pakistan.

## METHODS

### Study setting and participants

This cross-sectional study was carried out in Islamabad, Pakistan, from June 2023 to March 2024. The sample was recruited using a convenience sampling method. Participants included young individuals aged 16–25 years residing in Islamabad who had been using electronic cigarettes for at least 12 months. Exclusion criteria encompassed individuals engaged in dual smoking (both cigarette smoking and vaping), those with systemic diseases such as acquired immunodeficiency syndrome, cardiovascular disorders, diabetes mellitus, etc., as well as those who had undergone any periodontal therapy within the last 12 months.

The sample size was determined using OpenEpi software (version 3.01) and was based on data from a previous study, indicating that 22.7% of e-cigarette users reported experiencing poor oral health, including reddish and/or swollen gums^15^. With a confidence level of 95% and a power of 80%, along with a ratio of 1, the minimum required sample size was calculated to be 136 individuals. To account for a 10% dropout rate, the final sample size was set at 156 young adults.

The study was approved by the Ethics Review Committee of the School of Dentistry, Shaheed Zulfiqar Ali Bhutto Medical University (Reference no. SOD/ERB/2023/054). Prior to their involvement in the study, all participants provided written informed consent after being briefed on the study’s objectives. Participants were also made aware of their right to withdraw from the research at any point.

All consenting patients were subjected to an intraoral examination and completed a self-administered questionnaire. A single investigator conducted all examinations in a room with the patient seated in a reclined chair under adequate lighting. The examination tools included a mouth mirror, explorer, and periodontal probe.

The Decayed, Missing, and Filled Teeth (DMFT) index was used to assess the dental caries experience of the participants^16^. The DMFT score for each participant was calculated by summing the total number of teeth that were decayed, missing due to caries, or filled. A higher DMFT score indicates a greater burden of dental caries. Oral hygiene status was assessed using the Oral Hygiene Index Simplified (OHI-S), developed by Greene and Vermillion^17^. The OHI-S measures the accumulation of debris and calculus on six specific index teeth. Each tooth surface was scored using a 0 to 3 scale for fraction covered by debris or calculus: 0, none; 1, less than one-third; 2, one-third to two-thirds; and 3, more than two-thirds. The scores for debris and calculus were combined to provide an overall OHI-S score for each participant. Based on the OHI-S score, participants were categorized into three groups to reflect their overall oral hygiene status^17^: good, 0.0–1.2; fair, 1.3–3.0; and poor, 3.1–6.0.

The bleeding of the gums was assessed using the Gingival Bleeding Index (GBI) developed by Ainamo and Bay^18^. This index evaluates gingival bleeding at six sites around each tooth (mesiobuccal, midbuccal, distobuccal, mesiolingual, midlingual, and distolingual) using a scoring system from 0 to 3, where: 0 indicates no bleeding, 1 indicates isolated bleeding spots, 2 indicates blood forming a confluent line along the gingival margin, and 3 indicates heavy or profuse bleeding. For analysis purposes, individuals were divided into two categories based on whether they exhibited gingival bleeding or not. GBI scores of 0 and 1 were grouped together to represent the absence or minimal gingival bleeding, while scores of 2 and 3 were grouped together to represent the presence of gingival bleeding^18^. This approach simplified the analysis by focusing on the presence or absence of gingival bleeding rather than its severity.

Plaque accumulation was assessed using the Silness and Löe Plaque Index^19^. This index evaluates the presence of dental plaque on four tooth surfaces (buccal, lingual, mesial, and distal) using a scoring system from 0 to 3, where 0 indicates no plaque, 1 indicates a film of plaque adhering to the tooth surface that can be detected by running a probe across the tooth surface, 2 indicates a moderate accumulation of plaque visible to the naked eye, and 3 indicates abundant plaque accumulation visible to the naked eye and covering most of the tooth surface. Similar to the approach used for GBI, individuals were divided into two categories based on whether they exhibited plaque accumulation. PI scores of 0 and 1 were grouped together to represent the absence or minimal plaque, while scores of 2 and 3 were grouped together to represent the presence of plaque. This approach simplified the analysis by focusing on the presence or absence of plaque rather than the severity of accumulation.

Dental stain was assessed using the Lobene Stain Index^20^. The index teeth selected for this study were the maxillary and mandibular incisors and canines, as these teeth are more prone to staining in cigarette smokers. Each tooth was divided into three regions: the gingival margin, the body of the tooth, and the interproximal region. For each region, the extent of staining was evaluated using a scoring system from 0 to 3, where 0 indicates no stain, 1 indicates a stain covering less than one-third of the surface, 2 indicates a stain covering one-third to two-thirds of the surface, and 3 indicates a stain covering more than two-thirds of the surface. The scores for each region were averaged to provide an overall stain score for each tooth, and subsequently, an overall stain score for each participant. Participants were then categorized based on their average scores into the following groups: no stain (average score of 0), mild/moderate stain (average score of 1 to 2), and severe stain (average score of 3). This method allowed for a quantitative assessment of extrinsic dental staining among vapers, facilitating the comparison of stain severity across participants.

After intraoral examination the participants were requested to complete a questionnaire comprising 19 items divided into four sections, adapted from Hang^21^ and Chaudhary et al.^14^. The first section gathered sociodemographic information, including age, gender, education level, and personal income (four items). The second section evaluated participants’ vaping experiences (four items: vaping frequency, age at the onset of vaping, source of vape supplies, reason for initiating vaping). The third section assessed participants’ self-perceived oral health status and oral health behaviors (five items: perception of tooth health, perception of gum health, frequency of tooth brushing, frequency of mouthwash usage, and frequency of dental check-ups). Finally, the fourth section inquired about the perceived risks of vaping on oral health (six items: impact of vaping on tooth decay, gum disease, teeth staining, dry mouth, bad breath, and the risk of oral cancer).

### Statistical analysis

The data analysis was conducted using the Statistical Package for the Social Sciences (IBM SPSS Statistics V.26) software, with a significance level set at 5%. Descriptive quantitative analyses were employed to analyze and summarize the survey data. No time limit was imposed on questionnaire completion. To assess associations between qualitative variables and electronic cigarette use, the chi-squared test and Fisher’s exact test were conducted as appropriate. For the continuous variable DMFT, correlation analyses were performed to evaluate its relationship with the variables.

## RESULTS

A total of 142 participants was included in this study. The demographic breakdown showed that the majority was aged 21–25 years (57.7%), and a higher proportion were male (76.1%). Most participants had attained a high school education or higher (86%). Regarding self-perceived oral health status, a majority of participants reported good dental health (80.3%) and periodontal health (71.8%). In terms of oral health behaviors, 76.1% of participants reported brushing their teeth daily. However, only 34.5% reported using mouthwash daily, and a similarly low percentage (34.5%) attended regular dental check-ups. Clinical dental health examinations revealed that the participants had a mean DMFT score of 5.66 (SD=2.20). Additionally, 29.6% of participants exhibited poor oral hygiene and severe staining on their teeth. Gingival bleeding was observed in 47.2% of participants, and plaque was present in 35.3% of the participants (Table 1).

**Table 1:** Sociodemographic characteristics of the young users of ECs, Islamabad, Pakistan (N=142)

*Characteristics*	*n (%)*
**Age** (years)	
16–20	60 (42.3)
21–25	82 (57.7)
**Gender**	
Male	108 (76.1)
Female	34 (23.9)
**Education level**	
Primary	20 (14.1)
High School	61 (43.0)
University	61 (43.0)
**Income (PKR)**	
<50000	81 (57.0)
50000–100000	32 (22.5)
>100000	29 (20.4)
**Vaping use per day**	
Non-daily	49 (34.5)
1–10	38 (26.8)
11–19	26 (18.3)
>19	29 (20.4)
**Months since started using vaping**	
Around 12	41 (28.9)
12–18	28 (19.7)
18–24	40 (28.2)
>24	33 (23.2)
**Self-perceived dental health**	
Good	114 (80.3)
Poor	28 (19.7)
**Self-perceived periodontal health**	
Good	102 (71.8)
Poor	40 (28.2)
**Frequency of daily tooth brushing**	
None	3 (2.1)
Once	108 (76.1)
≥2 times	31 (21.8)
**Frequency of mouthwash use**	
None	80 (56.3)
Everyday	49 (34.5)
Sometime only	13 (9.2)
**Regular dental check-up**	
Yes	49 (34.5)
No	93 (65.5)
**DMFT**, mean (SD)	5.66 (2.20)
**OHI-S**	
Good	47 (33.1)
Fair	53 (37.3)
Poor	42 (29.6)
**Stain**	
No stain	59 (41.5)
Mild/moderate	41 (28.9)
Severe	42 (29.6)
**Gingival bleeding**	
Absence	75 (52.8)
Presence	67 (47.2)
**Plaque**	
Absence	92 (64.8)
Presence	50 (35.2)

DMFT: decayed, missing, and filled teeth. OHI-S: oral hygiene index simplified. PKR: 1000 Pakistani Rupees about US$3.6.

The study results on vaping practices among participants are presented in Table 2. A majority (66.2%) reported using vaping or electronic cigarettes daily. Notably, 80.3% of participants began vaping before the age of 18 years. The primary sources for obtaining vape supplies were online suppliers and stores (57.0%). When asked about their initial reasons for trying vaping, the most common response (31.7%) was the perception that vaping and e-cigarettes are safer than smoking.

**Table 2:** Questionnaire responses regarding vaping practice, among young users of ECs, Islamabad, Pakistan (N=142)

*Vaping practice*	*n (%)*
**How old were you when you started vaping?** (years)	
<18	114 (80.3)
≥18	28 (19.7)
**Where did/do you get your vape supplies from?**	
From family members	6 (4.2)
From friends	26 (18.3)
Online from Internet suppliers/stores	81 (57.0)
From a vape specialty store	29 (20.4)
Other (please specify)	-
**When you first tried vaping, what were the reasons for trying?**	
My friends or family members do it	22 (15.5)
Flavors are good	14 (9.9)
It’s safer than smoking	45 (31.7)
Wanted to quit smoking	28 (19.7)
Vaping ads made me want to try it	3 (2.1)
It’s cool	13 (9.2)
As a way of coping with stress	10 (7.0)
I wanted to know what it was like	7 (4.9)
Other (please specify)	-

The study results regarding participants’ perceptions of oral health risks of vaping are presented in Table 3. A substantial number of participants believed that vaping can contribute to tooth decay (44.4%) and is related to gum disease (47.9%). Additionally, a higher percentage of participants (58.5%) thought that vaping can lead to teeth staining, and 45.1% believed it contributes to bad breath. Conversely, a majority of participants (51.4%) did not think that vaping increases the risk of oral cancer. Regarding the contribution of vaping to dry mouth, most participants were unsure (38.0%).

**Table 3:** Perceptions of oral health risks of vaping, among young users of ECs, Islamabad, Pakistan (N=142)

*Vaping and oral health*	*n (%)*
**Do you think vaping can contribute to tooth decay?**	
Yes	63 (44.4)
No	59 (41.5)
Unsure	20 (14.1)
**Do you think vaping is related to gum disease?**	
Yes	68 (47.9)
No	50 (35.2)
Unsure	24 (16.9)
**Do you think vaping can contribute to teeth staining?**	
Yes	83 (58.5)
No	41 (28.9)
Unsure	18 (12.7)
**Do you think vaping can contribute to dry mouth?**	
Yes	40 (28.3)
No	48 (33.8)
Unsure	54 (38.0)
**Do you think vaping can contribute to bad breath?**	
Yes	64 (45.1)
No	47 (33.1)
Unsure	31 (21.8)
**Do you think vaping increases the risk of oral cancer?**	
Yes	45 (31.7)
No	73 (51.4)
Unsure	24 (16.9)

The analysis of sociodemographic factors, self-perceived oral health status, and clinical indicators (DMFT, OHI-S, stains, gingival bleeding, and plaque index) revealed significant associations with tooth brushing frequency, vaping frequency (per day), and time since vaping started (p<0.05). Stains and gingival bleeding were also significantly associated with regular dental check-ups (p<0.05). DMFT scores were notably lower among non-daily vapers (mean=4.2, SD=1.76) compared to those vaping over 19 times daily (mean=8.2, SD=0.95). Participants who started vaping ≤12 months ago had a lower DMFT (mean=3.2, SD=0.94) compared to those vaping for over two years (mean=8.2, SD=1.08). Additionally, participants brushing two or more times daily had a significantly lower DMFT (mean=3.6, SD=1.57) compared to those not brushing regularly (mean=7.6, SD=3.51) (Table 4).

**Table 4:** The association between sociodemographic characteristics, self-perceived oral health status and behaviors, and clinical oral health status indicators, among young users of ECs, Islamabad, Pakistan (N=142)

*Characteristics*	*DMFT* *Mean (SD)*	*OHI-S* *n (%)*			*Stain* *n (%)*			*Gingival bleeding* *n (%)*		*Plaque* *n (%)*	
		*Good*	*Fair*	*Poor*	*No stain*	*Mild/moderate*	*Severe*	*Absence*	*Presence*	*Absence*	*Presence*
**Age** (years)											
16–20	5.5 (1.91)	18 (38.3)	23 (43.4)	19 (45.2)	24 (40.7)	13 (31.7)	23 (54.8)	30 (40.0)	30 (44.8)	36 (39.1)	24 (48.0)
21–25	5.7 (2.40)	29 (61.7)	30 (56.6)	23 (54.8)	35 (59.3)	28 (68.3)	19 (45.2)	45 (60.0)	37 (55.2)	56 (60.9)	26 (52.0)
p	0.661	0.785			0.099			0.343		0.199	
**Gender**											
Male	5.7 (2.22)	37 (78.7)	41 (77.4)	30 (71.4)	44 (74.6)	34 (82.9)	30 (76.1)	58 (77.3)	50 (76.1)	71 (77.2)	37 (74.0)
Female	5.3 (2.15)	10 (21.3)	12 (22.6)	12 (28.6)	15 (25.4)	7 (17.1)	12 (28.6)	17 (22.7)	17 (25.4)	21 (22.8)	13 (26.0)
p	0.351	0.695			0.443			0.428		0.410	
**Education level**											
Primary	6.0 (2.17)	5 (10.6)	10 (18.9)	5 (11.9)	9 (15.3)	7 (17.1)	4 (9.5)	11 (14.7)	9 (13.4)	12 (13.0)	8 (16.0)
High School	6.1 (2.01)	18 (38.3)	21 (39.6)	22 (52.4)	23 (39.0)	15 (36.6)	23 (54.8)	31 (41.3)	30 (44.8)	42 (45.7)	19 (38.0)
University	5.0 (2.29)	24 (51.1)	22 (41.5)	15 (35.7)	27 (45.8)	19 (46.3)	15 (35.7)	33 (44.0)	28 (41.8)	38 (41.3)	23 (46.0)
p	0.023	0.420			0.458			0.916		0.667	
**Income** (PKR)											
<50000	5.6 (2.26)	25 (53.2)	33 (62.3)	23 (54.8)	35 (59.3)	23 (56.1)	23 (54.8)	43 (57.3)	38 (56.7)	53 (57.6)	28 (56.0)
50000–100000	5.6 (2.06)	12 (25.5)	9 (17.0)	11 (26.2)	10 (16.9)	8 (19.5)	14 (33.3)	15 (20.0)	17 (25.4)	21 (22.8)	11 (22.0)
>100000	5.6 (2.27)	10 (21.3)	11 (20.8)	8 (19.0)	14 (23.7)	10 (24.4)	5 (11.9)	17 (22.7)	12 (17.9)	18 (19.6)	11 (22.0)
p	0.984	0.803			0.251			0.655		0.942	
**Vaping use per day**											
Non-daily	4.2 (1.76)	28 (56.0)	15 (30.0)	7 (14.0)	32 (64.0)	9 (18.0)	9 (18.0)	35 (70.0)	15 (30.0)	39 (78.0)	11 (22.0)
1–10	4.7 (1.74)	16 (43.2)	16 (43.2)	5 (13.5)	22 (59.5)	13 (35.1)	2 (5.4)	27 (73.0)	10 (27.0)	29 (78.4)	8 (21.6)
11–19	6.9 (1.01)	3 (11.5)	13 (50.0)	10 (38.5)	3 (11.5)	14 (53.8)	9 (34.6)	10 (38.5)	16 (61.5)	15 (57.7)	11 (42.3)
>19	8.2 (0.95)	0	9 (31.0)	20 (69.0)	2 (6.9)	5 (17.2)		3 (10.3)	26 (89.7)	9 (31.0)	20 (69.0)
p	**0.001**	**0.001**			**0.001**			**0.001**		**0.001**	
**Months since started vaping**											
Around 12	3.2 (0.94)	29 (70.7)	11 (26.8)	1 (2.4)	40 (97.6)	0		36 (87.8)	5 (12.2)	37 (90.2)	4 (9.8)
12–18	4.8 (1.36)	11 (39.3)	11 (39.3)	6 (21.4)	10 (35.7)	11 (39.3)	7 (25.0)	17 (60.7)	11 (39.3)	20 (71.4)	8 (28.6)
18–24	6.6 (1.21)	6 (15.0)	22 (55.0)	12 (30.0)	8 (20.0)	20 (50.0)	12 (30.0)	20 (50.0)	20 (50.0)	29 (72.5)	11 (27.5)
>24	8.2 (1.08)	1(3.0)	9 (27.3)	23 (69.7)	1 (3.0)	10 (30.3)	22 (66.7)	2 (6.1)	31 (93.9)	6 (18.2)	27 (81.8)
p	**0.001**	**0.001**			**0.001**			**0.001**		**0.001**	
**Self-perceived dental health**											
Good	5.5 (2.10)	38 (80.9)	46 (86.8)	30 (71.4)	49 (83.1)	37 (90.2)	28 (66.7)	63 (84.0)	51 (76.1)	76 (82.6)	38 (76.0)
Poor	5.9 (2.58)	9 (19.1)	7 (13.2)	12 (28.6)	10 (16.9)	4 (9.8)	14 (33.3)	12 (16.0)	16 (23.9)	16 (17.4)	12 (24.0)
p	0.420	0.173			**0.021**			0.167		0.233	
**Self-perceived periodontal health**											
Good	5.6 (2.15)	35 (74.5)	39 (73.6)	28 (66.7)	44 (74.6)	33 (80.5)	25 (59.5)	56 (74.7)	46 (68.7)	66 (71.7)	36 (72.0)
Poor	5.8 (2.35)	12 (25.5)	14 (26.4)	14 (33.3)	15 (25.4)	8 (19.5)	17 (40.5)	19 (25.3)	21 (31.3)	26 (28.3)	14 (28.0)
p	0.642	0.672			0.087			0.271		0.568	
**Frequency of tooth brushing**											
None	7.6 (3.51)	1 (2.1)	0	2 (4.8)	1 (1.7)	0	2 (4.8)	1 (1.3)	2 (3.0)	1 (1.1)	2 (4.0)
Once	6.1 (1.99)	27 (57.4)	42 (79.2)	39 (92.9)	33 (55.9)	37 (90.2)	38 (90.5)	48 (64.0)	60 (89.6)	65 (70.7)	43 (86.0)
≥2 times	3.6 (1.57)	19 (40.4)	11 (20.8)	1 (2.4)	25 (42.4)	4 (9.8)	2 (4.8)	26 (34.7)	5 (7.5)	26 (28.3)	5 (10.0)
p	**0.001**	**0.001**			**0.001**			**0.001**		**0.027**	
**Frequency of mouthwash use**											
None	5.8 (2.19)	27 (57.4)	28 (52.8)	25 (59.5)	30 (50.8)	23 (56.1)	27 (64.3)	39 (52.0)	41 (61.2)	51 (55.4)	29 (58.0)
Everyday	5.6 (2.24)	15 (31.9)	19 (35.8)	15 (35.7)	20 (33.9)	15 (36.6)	14 (33.3)	25 (33.3)	24 (35.8)	30 (32.6)	19 (38.0)
Sometime only	4.9 (2.13)	5 (10.6)	6 (11.3)	2 (4.8)	9 (15.3)	3 (7.3)	1 (2.4)	11 (14.7)	2 (3.0)	11 (12.0)	2 (4.0)
p	0.413	0.807			0.238			0.053		0.279	
**Regular dental check-up**											
Yes	5.1 (2.17)	19 (40.4)	20 (37.7)	10 (23.8)	26 (44.1)	16 (39.0)	7 (16.7)	32 (42.7)	17 (25.4)	34 (37.0)	15 (30.0)
No	5.9 (2.18)	28 (59.6)	33 (62.3)	32 (76.2)	33 (55.9)	25 (61.0)	35 (83.3)	43 (57.3)	50 (74.6)	58 (63.0)	35 (70.0)
p	**0.049**	0.212			**0.013**			**0.023**		0.260	

DMFT: decayed, missing, and filled teeth. OHI-S: oral hygiene index simplified. PKR: 1000 Pakistani Rupees about US$3.6.

The analysis of the association between perceptions of oral health risks of vaping and sociodemographic characteristics revealed that education level is the only variable significantly associated with all vaping-related oral health risk perception questions (p<0.05). Participants with higher levels of education were more likely to believe that vaping contributes to various oral health issues: tooth decay (65.1%), gum disease (60.3%), teeth staining (53.0%), dry mouth (67.5%), bad breath (60.9%), and an increased risk of oral cancer (60.0%) (Table 5).

**Table 5:** The association between perception of oral health risks of vaping and sociodemographic characteristics, among young users of ECs, Islamabad, Pakistan (N=142)

*Vaping and oral health*	*Age (years)* *n (%)*		*Gender* *n (%)*		*Education level* *n (%)*			*Income (PKR)* *n (%)*		
	*16–20*	*21–25*	*Male*	*Female*	*Primary*	*High school*	*University*	*<50000*	*50000–100000*	*>100000*
**Do you think vaping can contribute to tooth decay?**										
Yes	26 (41.3)	37 (58.7)	47 (74.6)	16 (25.4)	3 (4.8)	19 (30.2)	41 (65.1)	33 (52.4)	15 (23.8)	15 (23.8)
No	25 (42.4)	34 (57.6)	46 (78.0)	13 (22.0)	11 (18.6)	34 (57.6)	14 (23.7)	35 (59.3)	13 (22.0)	11 (18.6)
Unsure	9 (45.0	11 (55.0)	15 (75.0)	5 (25.0)	6 (30.0)	8 (40.0)	6 (30.0)	13 (65.0)	4 (20.0)	3 (15.0)
p	0.95		0.90		**0.001**			0.92		
**Do you think vaping is related to gum disease?**										
Yes	28 (41.2)	40 (58.8)	51 (75.0)	17 (25.0)	5 (7.4)	22 (32.4)	41 (60.3)	39 (57.4)	14 (20.6)	15 (22.1)
No	21 (42.0)	29 (58.0)	38 (76.0)	12 (24.0)	9 (18.0)	29 (58.0)	12 (24.0)	29 (58.0)	11 (22.0)	10 (20.0)
Unsure	11 (45.8)	13 (54.2)	19 (79.2)	5(20.8)	6 (25.0)	10 (41.7)	8 (33.3)	13 (54.2)	7 (29.2)	4 (16.7)
p	0.92		0.91		**0.001**			0.93		
**Do you think vaping can contribute to teeth staining?**										
Yes	35 (42.2)	48 (57.8)	63 (75.9)	20 (24.1)	9 (10.8)	30 (36.1)	44 (53.0)	46 (55.4)	19 (22.9)	18 (21.7)
No	17 (41.5)	24 (58.5)	31 (75.6)	10 (24.4)	7 (17.1)	24 (58.5)	10 (24.4)	25 (61.0)	9 (22.0)	7 (17.1)
Unsure	8 (44.4)	10 (55.6)	14 (77.8)	4 (22.2)	4 (22.2)	7 (38.9)	7 (38.9)	10 (55.6)	4 (22.2)	4 (22.2)
p	0.97		0.98		**0.035**			0.97		
**Do you think vaping can contribute to dry mouth?**										
Yes	20 (50.0)	20 (50.0)	29 (72.5)	11 (27.5)	3 (7.5)	10 (25.0)	27 (67.5)	17 (42.5)	11 (27.5)	12 (30.0)
No	21 (43.8)	27 (56.3)	38 (79.2)	10 (20.8)	9 (18.8)	26 (54.2)	13 (27.1)	28 (58.3)	9 (18.8)	11 (22.9)
Unsure	19 (35.2)	35 (64.8)	41 (75.9)	13 (24.1)	8 (14.8)	25 (46.3)	21 (38.9)	36 (66.7)	12 (22.2)	6 (11.1)
p	0.34		0.76		**0.004**			0.11		
**Do you think vaping can contribute to bad breath?**										
Yes	25 (39.1)	39 (60.90	47 (73.4)	17 (26.6)	5 (7.8)	20 (31.1)	39 (60.9)	35 (54.7)	14 (21.9)	15 (23.4)
No	20 (42.6)	27 (57.4)	35 (74.5)	12 (25.5)	7 (14.9)	27 (57.4)	13 (27.7)	29 (61.7)	10 (21.3)	8 (17.0)
Unsure	15 (48.4)	16 (51.6)	26 (83.9)	5 (16.1)	8 (25.8)	14 (45.2)	14 (45.2)	17 (54.8)	8 (25.8)	6 (19.4)
p	0.68		0.51		**0.001**			0.90		
**Do you think vaping increases risk of oral cancer?**										
Yes	19 (42.2)	26 (57.8)	32 (71.1)	13 (28.9)	3 (6.7)	15 (33.3)	27 (60.0)	22 (48.9)	10 (22.2)	13 (28.9)
No	32 (43.8)	41 (56.2)	58 (79.5)	15 (20.5)	11 (15.1)	37 (50.7)	25 (34.2)	44 (60.3)	17 (23.3)	12 (16.4)
Unsure	9 (37.5)	15 (62.5)	18 (75.0)	6 (25.0)	6 (25.0)	9 (37.5)	9 (37.5)	15 (62.5)	5 (20.8)	4 (16.7)
p	0.86		0.58		**0.031**			0.53		

PKR: 1000 Pakistani Rupees about US$3.6.

## DISCUSSION

This study aimed to assess the oral health status, perceptions of vaping, and associated oral health risks among young adults in Pakistan. The findings provide valuable insights that could support local and national initiatives focused on reducing vaping adoption among non-smokers and current vapers. The study sample comprised predominantly males (76.1%), which aligns with similar studies conducted in Indonesia, South Korea, and Canada, where e-cigarette use is also dominated by men^11,22,23^. Studies from countries like Peru have reported even lower percentages of female EC smokers^15^. This trend may be attributed to the traditionally low prevalence of conventional cigarette use among women, which appears to extend to electronic cigarette use as well.

In terms of self-perceived oral health, a significant majority of participants (80.1% for dental health and 71.8% for periodontal health) reported good status. These findings are comparable to a study in the United States where most participants rated their periodontal status as moderate to good on a 10-point scale^24^. The relatively young age of our study population (aged <25 years) may explain the lower incidence of periodontal diseases, which are typically more prevalent in adults aged 35–74 years.

Despite these positive self-assessments, nearly half of the participants recognized the potential risks of vaping on oral health, with 47.9% acknowledging its contribution to tooth decay and 44.4% to gum disease. Existing literature supports these concerns, demonstrating associations between electronic nicotine use and periodontal disease^12,15^. For instance, menthol flavoring in e-cigarette liquids has been shown to reduce the proliferation rate of human periodontal ligament fibroblasts^13^. Nicotine present in e-cigarette aerosols can inhibit the growth of gingival fibroblasts and periodontal ligament cells, alter neutrophil function, promote oral inflammation, and accelerate senescence of periodontal fibroblasts^13^. Additionally, propylene glycol, a major component of e-liquids, may decrease tooth integrity by altering calcium release and tooth mineralization. E-cigarette use has also been linked to inflammation, oxidative stress, impaired host response, and dysregulated repair mechanisms, all contributing to periodontal disease and poor oral hygiene^25^.

The study reported an average DMFT score of 5.66 among participants, which is comparable to the score of 7.06 observed in young vapers in Indonesia^11^. Similar studies conducted in France, the USA, and Malaysia have also indicated higher DMFT scores among e-cigarette users compared to non-users^26-28^. Nicotine’s role in enhancing *Streptococcus mutans* biofilm formation and metabolic activity is well-documented, leading to increased caries development. The acid production from these biofilms lowers the local pH, promoting demineralization of enamel and dentin^29^.

Regarding dental staining, approximately 60% of participants exhibited mild to severe tooth stains. Correspondingly, a study among young Indonesian vapers found that 33.33% had heavy staining, 53.33% had moderate staining, and 13.33% had no staining^11^. Other studies have also reported a high prevalence of dry mouth among e-cigarette users^11,30^. However, in our study, a vast majority (71.8%) of participants were either unaware or did not believe that vaping could contribute to dry mouth. Similar trends were observed regarding perceptions of the contribution of vaping to tooth decay, gum disease, bad breath, and oral cancers. This indicates a need for increased awareness about the comprehensive oral health risks associated with e-cigarette use, as studies have linked vaping to various oral mucosal conditions, including xerostomia (dry mouth) and hairy tongue^26,31^.

Encouragingly, most participants demonstrated good oral hygiene practices, with over 70% reporting daily tooth brushing and achieving good to fair oral hygiene index scores. These results are consistent with a study conducted in Portugal, where the majority of young vapers also maintained good oral hygiene^32^. This may be attributed to the high level of education among participants (86% with higher school or university education), leading to greater awareness and knowledge about the importance of oral hygiene. Notably, education level was the only sociodemographic factor significantly associated with perceptions of oral health risks of vaping, in the present study.

The study also revealed concerning patterns in vaping practices. A majority (66%) of participants used e-cigarettes daily, and alarmingly, over 80% began vaping before the age of 18 years. Similar trends have been observed in Saudi Arabia and other countries, indicating that e-cigarette use has become a popular habit among young students^33,34^. The primary reason cited for initiating vaping was the perception that it is safer than conventional smoking. This misconception is likely fueled by aggressive marketing campaigns targeting youth, promoting vaping as a safer alternative and a lifestyle choice^35,36^. The ease of access to e-cigarette supplies through online stores further facilitates this trend, highlighting the need for stricter regulations and enforcement to control underage access^35,36^.

### Limitations

This study, while providing important initial insights into the oral health implications of vaping among Pakistani youth, has several limitations. Social desirability bias may have influenced self-reported data, with participants potentially underreporting or altering their experiences. The generalizability of the results is limited due to the unrepresentative sample concerning all age groups and geographical locations within Pakistan. Additionally, the cross-sectional design of the study does not allow for establishing causal relationships between e-cigarette use and oral health outcomes. Another limitation is the potential for residual confounding, as unmeasured or unknown factors may have influenced the observed associations between electronic cigarette use and oral health outcomes. Additionally, the lack of adjusted comparisons limits our ability to draw definitive causal inferences. Although we performed association analyses, the absence of multivariable adjustments restricts our understanding of the independent effects of specific variables on oral health status. Future studies incorporating a larger sample size and more comprehensive adjustment for potential confounders would enhance the robustness of the findings.

### Implications for policy and regulation

Despite the limitations, the study’s findings have significant implications for policy and regulation. There is a pressing need for comprehensive vaping policies in Pakistan that adopt a precautionary approach, acknowledging the scientific uncertainty surrounding long-term health consequences and focusing on reducing and prohibiting vaping, especially among youth. Integrating information about the harmful effects of e-cigarettes into dental education curricula and training dental professionals to support cessation efforts are critical steps forward. Dental professionals are uniquely positioned to counsel individuals on the risks of vaping and participate actively in anti-smoking campaigns and community outreach programs to enhance awareness and promote oral health education.

## CONCLUSIONS

This study provides valuable insights into the oral health perceptions and practices among young vapers in Pakistan, highlighting both the self-perceived and clinically observed effects of vaping on oral health. The findings show that awareness regarding the oral health risks of vaping remains low, with a significant number of users starting to vape before the age of 18 years. A prevalent misconception that e-cigarettes are less harmful than traditional cigarettes appears to be a key driver behind their use. Participants exhibited poor dental health, as reflected in their DMFT scores, poor oral hygiene, gingival bleeding, and overall oral health behaviors. The results suggest that although young vapers are aware of some oral health risks associated with vaping, particularly tooth decay and gum disease, a lack of awareness persists regarding other potential risks, such as dry mouth and oral cancer. Education level emerged as a significant factor in shaping these perceptions, emphasizing the need for targeted public health campaigns to increase awareness of the full spectrum of vaping on oral health risks.

## Data Availability

The data supporting this research are available from the authors on reasonable request.
